# Lymph node-targeting adjuvant/neoantigen-codelivering vaccines for combination glioblastoma radioimmunotherapy

**DOI:** 10.7150/thno.84443

**Published:** 2023-08-06

**Authors:** Ting Su, Shurong Zhou, Suling Yang, Nicholas Humble, Fuwu Zhang, Guocan Yu, Paula D. Bos, Furong Cheng, Kristoffer Valerie, Guizhi Zhu

**Affiliations:** 1 Department of Pharmaceutics and Center for Pharmaceutical Engineering and Sciences; The Developmental Therapeutics Program, Massey Cancer Center; Virginia Commonwealth University, Richmond, VA 23298, USA.; 2 Department of Radiation Oncology, School of Medicine; The Developmental Therapeutics Program Program, Massey Cancer Center; Virginia Commonwealth University, Richmond, VA 23298, USA.; 3 Department of Chemistry, University of Miami, Coral Gables, FL 33146, USA.; 4 Key Laboratory of Bioorganic Phosphorus Chemistry & Chemical Biology, Department of Chemistry, Tsinghua University, Beijing, 100084, China.; 5 Department of Pathology, School of Medicine; Cancer Biology Program, Massey Cancer Center; Virginia Commonwealth University, Richmond, VA 23298, USA.; 6 Department of Pharmaceutical Sciences, College of Pharmacy; Biointerfaces Institute. University of Michigan, Ann Arbor, MI 48109, USA.

**Keywords:** albumin, DNA engineering, neoantigen vaccine, vaccine codelivery, glioblastoma immunotherapy

## Abstract

Glioblastoma multiforme (GBM) is the most common and lethal type of adult brain cancer. Current GBM standard of care, including radiotherapy, often ends up with cancer recurrence, resulting in limited long-term survival benefits for GBM patients. Immunotherapy, such as immune checkpoint blockade (ICB), has thus far shown limited clinical benefit for GBM patients. Therapeutic vaccines hold great potential to elicit anti-cancer adaptive immunity, which can be synergistically combined with ICB and radiotherapy. Peptide vaccines are attractive for their ease of manufacturing and stability, but their therapeutic efficacy has been limited due to poor vaccine co-delivery and the limited ability of monovalent antigen vaccines to prevent tumor immune evasion. To address these challenges, here, we report GBM radioimmunotherapy that combines radiotherapy, ICB, and multivalent lymph-node-targeting adjuvant/antigen-codelivering albumin-binding vaccines (AAco-AlbiVax). Specifically, to codeliver peptide neoantigens and adjuvant CpG to lymph nodes (LNs), we developed AAco-AlbiVax based on a Y-shaped DNA scaffold that was site-specifically conjugated with CpG, peptide neoantigens, and albumin-binding maleimide-modified Evans blue derivative (MEB). As a result, these vaccines elicited antitumor immunity including neoantigen-specific CD8^+^ T cell responses in mice. In orthotopic GBM mice, the combination of AAco-AlbiVax, ICB, and fractionated radiation enhanced GBM therapeutic efficacy. However, radioimmunotherapy only trended more efficacious over radiotherapy alone. Taken together, these studies underscore the great potential of radioimmunotherapy for GBM, and future optimization of treatment dosing and scheduling would improve the therapeutic efficacy.

## Introduction

GBM is the most common and lethal primary malignancy in adult central nervous system (CNS). Current standard-of-care GBM treatment involves surgery followed by chemoradiation, but tumors, especially GBMs with unmethylated O^6^-methylguanine DNA methyltransferase (MGMT) that resist to temozolomide chemotherapy, often relapse or progress 1,2. The CNS is immunologically unique due to the blood brain barrier (BBB) and blood tumor barrier (BTB), lack of conventional lymphatics, paucity of APCs, and low basal expression of major histocompatibility complex (MHC) molecules in CNS cells; yet, peripheral leukocytes access the brain and elicit robust immune responses under both inflammatory conditions and preclinical and clinical GBM immunotherapy studies 3,4. Immunotherapies that have proven effective in a growing number of cancers, such as ICB and chimeric antigen receptor T (CAR-T) adoptive cell therapy as well as therapeutic vaccines, have also been studied in GBM, but clinical outcomes have been mixed 5,6.

Encouraging examples of GBM immunotherapy include intracranial administration of CAR-T cells that regressed GBM tumors^7^,^8^; intratumoral infusion of oncolytic viruses that showed remarkable therapy efficacy in recurrent GBM 9,10; neoadjuvant systemic ICB using program death receptor 1 (PD-1) antibody (αPD-1) prior to surgery which promoted patient survival in recurrent GBM^11^,^12^; and multi-epitope neoantigen vaccines that have shown promising therapeutic efficacy in glioblastoma patients and remodeled GBM immune milieu 13-15. However, overall, GBM patients have responded poorly to current immunotherapies 16, largely due to the local and systemic immunosuppression in GBM patients 17, tumor heterogeneity and instability which is associated with the failure of a phase III clinical trial of a epidermal growth factor receptor variant III (EGFRvIII) vaccines 16,18, central immune tolerance against conventional tumor-associated vaccines, low tumor mutation burdens, and poor immunogenicity of the majority of tumor neoepitopes 19-21.

We attempted to address the above challenges by combining radiotherapy and immunotherapy, which hold the potential to be synergistically combined to elicit potent and durable anti-GBM immunity, maximizing GBM therapeutic efficacy. Firstly, conformal radiation of GBM tumors can selectively kill tumor cells but not healthy cells, but it can also mitigate immunosuppression, and disrupt BBB to promote intracranial infiltration of systemic lymphocytes^22^. Supporting our rational, preclinical studies have suggested that anti-GBM immunity can be restored by combining immunotherapy with radiotherapy, some of which have been tested in clinic (NCT02648633, NCT02866747) 23-25. Secondly, cancer-cell-specific neoantigens have been extensively explored as cancer therapeutic vaccines, and have shown remarkable efficacy in a number of human cancers, including GBM 13. Since spontaneous neoantigen-specific cytotoxic T lymphocytes (CTLs) are extremely rare 26, neoantigen vaccines can supply exogenous neoantigens to potentiate and expand neoantigen-specific CTLs. Lastly, rational combination of vaccines with ICB have been shown to synergistically reinvigorate anergic antitumor immunity and further improve cancer therapeutic efficacy 27.

Importantly, in this study we developed a LN-targeting DNA-scaffolded adjuvant/neoantigen co-delivering vaccines for GBM combination radioimmunotherapy. While conventional subunit vaccines have limited efficacy due to unfavorable pharmacokinetics, nanovaccines can be efficiently delivered to lymphoid tissues and APCs 28-31. Previously, we developed albumin/vaccine nanocomplexes that are self-assembled *in vivo* from endogenous albumin and a form of albumin-binding vaccines (AlbiVax) 32. Endogenous albumin is abundant and stable in the lymphatic system (t_1/2_: ~20 d in human) 32. AlbiVax are modular molecular conjugates of albumin-binding Evans blue derivative and molecular vaccines, including peptide antigens or molecular adjuvants (e.g., Toll-like receptor 9 (TLR9) agonist CpG oligonucleotide), separately. The resulting albumin/AlbiVax nanocomplexes of CpG and peptide antigens can be delivered to LNs up to ~100 times more efficiently than a clinic benchmark, incomplete Freund's adjuvant (IFA). Therefore, AAco-AlbiVax may address several limitations of peptide vaccines. First, antigen/adjuvant codelivery to LN APCs potentiates antigen immunogenicity and minimizes the immune tolerance caused by nonadjuvanted antigens or antigen presentation to T cells, thereby potentiating tumor therapeutic efficacy 33-35. Adjuvant/antigen codelivery 36,37 into LNs and APCs 37-44 allows the adjuvant to activate innate immunity and potentiate antigen immunogenicity, thereby promoting adaptive immunity 37. Second, given the neoantigen heterogeneity of GBM and the associated tumor immune escape that evade antitumor immunity, multi-antigen vaccines elicit broad antitumor immunity to enhance therapeutic efficacy 45,46. Third, the modular AAco-AlbiVax can be widely applicable to heterogeneous antigens to overcome tumor heterogeneity and the human leukocyte antigen (HLA) restriction of peptide vaccines in future clinical testing and application 47.

## Results and Discussion

### Design and synthesis of AAco-AlbiVax

Neoantigen vaccines and neoantigen-specific T cell receptor (TCR) adoptive T cell therapy have shown great potential for the immunotherapy of a growing number of cancers. Human GBM have shown significant somatic mutation burdens,^48^ suggesting the potential to develop immunogenic neoantigen vaccines to elicit GBM-specific T cell responses for immunotherapy, as validated in a Phase Ib clinical trial 13. For preclinical studies, immunogenic neoantigens have been previously identified in murine GBM cell lines, such as GL261^49^, and have been validated as vaccine targets that elicit potent GL261-specific T cell responses for GBM immunotherapy.^50^ Therefore, we used these three neoantigens, namely Ntrk1, Rtn2, and Imp3, all of which are MHC-I-restricted epitopes that would elicit CD8^+^ T cell responses. Peptide antigens are often poorly immunogenic, which requires the use of immunostimulatory adjuvants to potentiate their associated T cell responses. In this study, we used phosphorothioate-modified CpG oligonucleotide as the adjuvant. CpG is a TLR9 agonist that has been shown to elicit potent proinflammatory responses in mice and in human 51. CpG has been used as an adjuvant in an FDA-approved HBV protein subunit vaccine, and has been extensively tested for cancer immunotherapy in the clinic.

**Scheme 1:**
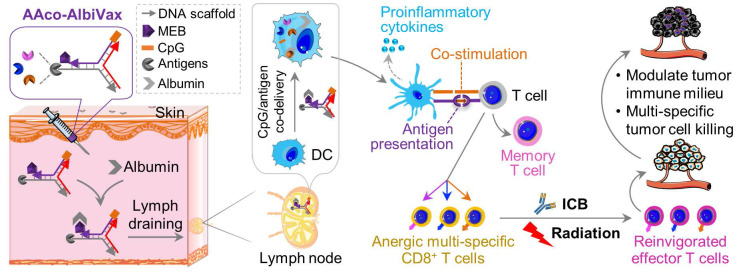
** Lymph-node-targeting AAco-AlbiVax for combination cancer radioimmunotherapy.** Upon *s.c.* administration, AAco-AlbiVax binds to endogenous albumin to form nanocomplexes, which are drained to LNs with prolonged retention. In LNs, AAco-AlbiVax are internalized by APCs, resulting in efficient presentation of antigenic epitopes, upregulated the expression of co-stimulation signals, and enhanced the production of proinflammatory cytokines, all of which are essential for T cell priming. As a result, AAco-AlbiVax, when combined with ICB and radiotherapy, improved the therapeutic efficacy of murine orthotopic GBM.

**Figure 1:**
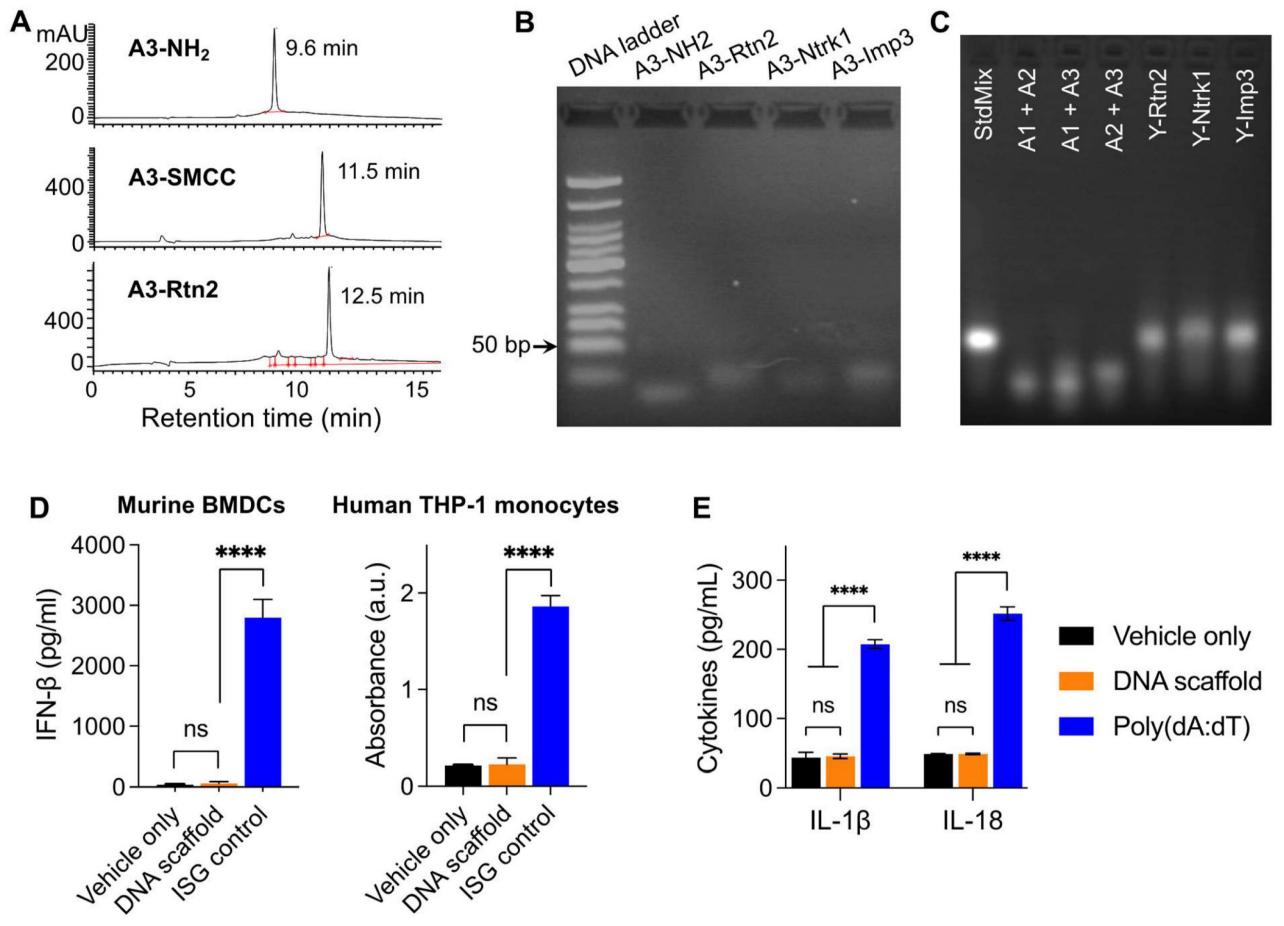
**Construction of Y-shaped DNA-scaffolded vaccines**. (A) Representative HPLC chromatograms of A3-NH_2_, A3-SMCC, and A3-Rtn2. (B) An agarose gel electrophoresis image showing the gel retarding of A3-Rtn2, A3-Ntrk1, and A3-Imp3 conjugates relative to A3-NH_2_, suggesting the successful conjugation of A3-NH_2_ with these neoantigen peptides. (C) An agarose gel electrophoresis image showing the formation of Y-shaped structures via the hybridization of A1-MEB, A2, with A3-Rtn, A3-Ntrk, and A3-Imp3, respectively. StdMix: hybridization of unmodified A1, A2, and A3; A1 + A2: mixed A1 and A2; A1 + A3: mixed A1 and A3; A2 + A3: mixed A2 and A3; Y-Rtn2: hybridized A1-MEB, A2, with A3-Rtn. Y-Ntrk1: hybridized A1-MEB, A2, with A3-Ntrk1. Y-Imp3: hybridized A1-MEB, A2, with A3-Imp3. (D) Negligible IFN-β responses by DNA scaffold in mouse BMDCs and human THP-1 monocytes (100 nM, 24 h). ISG DNA served as a positive control. (E) IL-1β and IL-18 responses showed negligible AIM2 activation by dsDNA scaffold in mouse BMDCs (100 nM, 24 h). Poly(dA:dT) dsDNA served as a positive control. Data: means ± SD. ns: non-significant; and ****p < 0.0001, by Two-way ANOVA. Transfection vehicle: lipofectamine 3000.

Because the codelivery of antigens and immunostimulatory adjuvants promotes antigen presentation and antigen-specific T cell responses, we designed a Y-shaped DNA scaffold that can be site-specifically modified with multiple functionalities (**Scheme 1**). Specifically, the Y-shaped DNA scaffold was designed to be formed via the hybridization of three 20-mer single-stranded DNA oligonucleotides, namely A1, A2, and A3 (see sequences in **Table S1**). A1 was modified with CpG on the 5' end which is expected to retain the immunostimulatory activity of CpG. We employed AlbiVax technology to promote vaccine delivery and retention to draining LNs which harbor a series of lymphocytes (e.g., APCs and naïve T cells) and coordinate adaptive immunomodulation. Specifically, we modified an albumin-binding MEB to 3'-end thiolate CpG-A1 to enable the resulting scaffolded vaccines to hitchhike endogenous albumin for LN homing and efficient APC uptake. The MEB-CpG-A1 conjugate was verified by HPLC, which revealed the characteristic UV absorption for DNA and MEB at the retention time of 11.51 min (**Fig. S1**). Furthermore, in MEB-CpG-A1, MEB showed negligible absorption at 260 nm relative to CpG-A1, which is the characteristic absorption wavelength of DNA, this allowed us to calculate MEB-CpG-A1 concentrations based on absorbance at 260 nm (**Fig. S2**). Further, to co-deliver CpG adjuvant and peptide neoantigens, 5'-end amine-modified A3 was conjugated with one of the three carboxylated peptide neoantigens (Ntrk1: CSSMSLQFMTL, Rtn2: CSSGAIFNGFTL, Imp3: CSSAALLNKLYA), by using succinimidyl-4-(N-maleimidomethyl)cyclohexane-1-carboxylate (SMCC) as a crosslinker. The A3 and neoantigen conjugation was purified by HPLC in which A3-peptide conjugate showed prolonged retention relative to A3-NH_2_ and A3-SMCC intermediate (**Fig. 1A**; **Fig. S3**). The A3-antigen conjugates were further verified by agarose gel electrophoresis which showed gel retardation of these conjugates relative to A3-NH_2_ (**Fig. 1B; Fig. S4**). Moreover, all these DNA scaffold as well as CpG was modified with DNase-resistant phosphorothioate backbone.^52^ Gel electrophoresis verified the good stability of Y-shaped DNA in 1% FBS-supplemented PBS at 37 °C (**Fig. S5**). Next, conjugates of A1, A2, and one of the three A3-antigens, respectively, were mixed at 1:1:1 molar ratio in PBS to be self-assembled into Y-shaped scaffolded vaccines. The resulting scaffolded vaccines were verified by gel electrophoresis (**Fig. 1C**). These results demonstrate the successful construction of Y-shaped DNA-scaffolded vaccines. Though long dsDNA (> 45 bp) has been shown to activate cytosolic DNA sensors cyclic GMP-AMP synthase (cGAS) and absent in melanoma 2 (AIM2) to induce innate immunity,^53^ our short dsDNA scaffold did not significantly activate cGAS (shown by interferon β (IFN-β)) (**Fig. 1D**) or AIM2 (shown by IL-1, IL18) (**Fig. 1E**) in mouse BMDCs and THP-1 human monocytes. This rules out the possibility of AAco-AlbiVax to elicit anti-scaffold immunity that would otherwise prevent repeated dosing.

### DC immunostimulation and antigen presentation by AAco-AlbiVax

**Figure 2:**
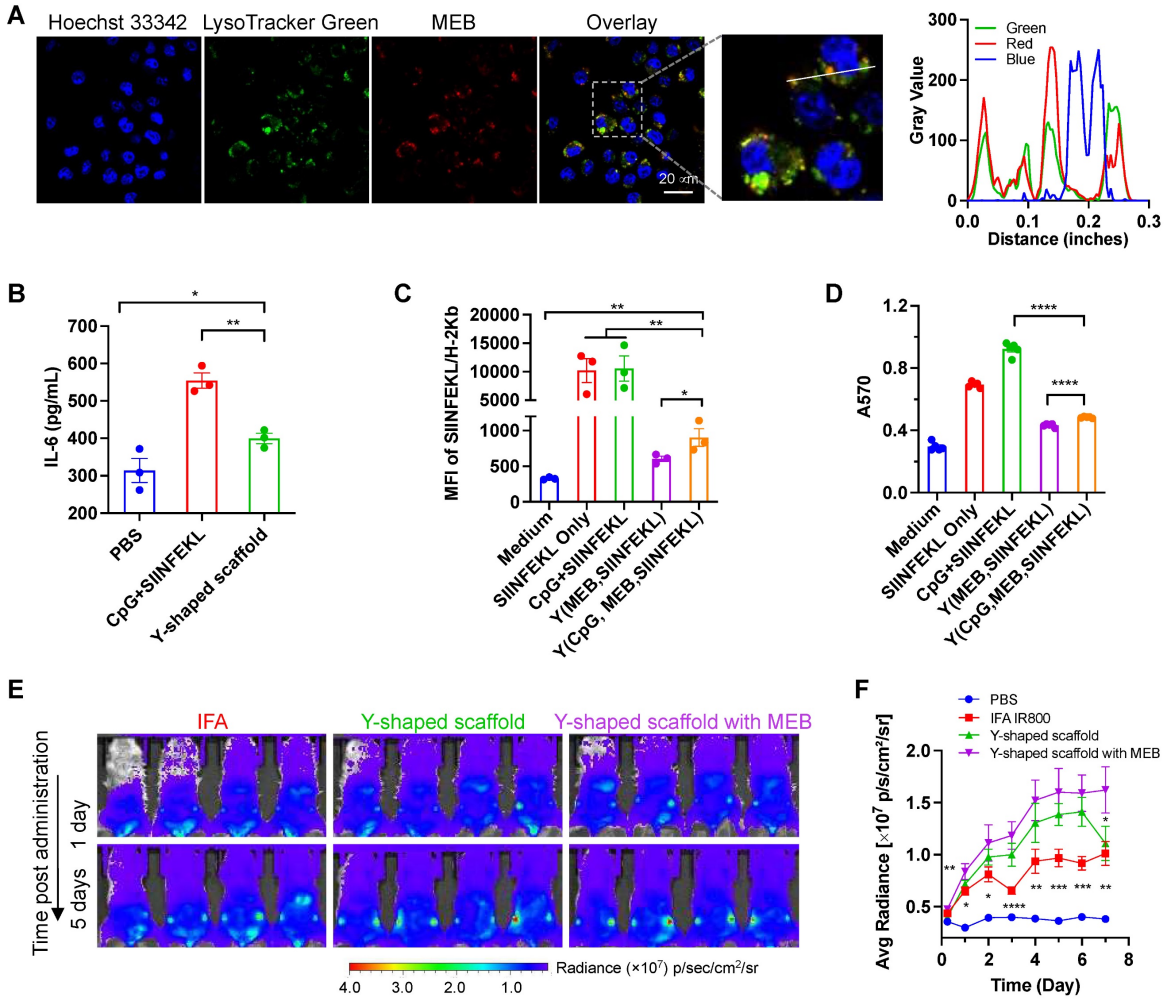
***In vitro* immunostimulation and *in vivo* LN delivery of AAco-AlbiVax.** (A) Confocal microscopy images showing the uptake of Y-shape scaffold into DC2.4 cells after a 3-h incubation. Scale bar: 20 µm. (B) ELISA results of IL-6 secretion from DC2.4 cells treated with free SIINFEKL + free CpG or Y-shape scaffold with CpG (100 nM) for 24 h. (C) Flow cytometric analysis of the SIINFEKL/H-2K^b^ complex levels on DC2.4 cells treated with SIIFENKL, free SIINFEKL + free CpG, or Y-shape scaffold with or without CpG, respectively, (CpG: 100 nM) for 24 h. (D) The activity of B3Z CD8^+^ T cells co-cultured with DCs pre-treated with the indicated vaccines. (E, F) Balb/c mice (n = 4) were s.c. injected (at the tail base) with IR800-labeled Y-shaped scaffold, with PBS, IR800 dye in IFA or Y-shape scaffold without MEB as controls, followed by IVIS imaging of mice. Shown are representative images illustrating the biodistribution at a series of time points post administration (E) and the quantified fluorescence signals in the same region of interest in draining inguinal LNs (F). Asterisks in (F) indicate statistical analysis between IFA IR800 or Y-shaped scaffold and Y-shaped scaffold with MEB. Data: means ± SEM. *p < 0.05, **p < 0.01, ***p < 0.001, and ****p < 0.0001 (Student's t-test).

The intracellular delivery of T cell vaccines to APCs are critical for their proteolytic processing (for antigenic peptide that are longer than minimal peptide epitopes), MHC complexation, and presentation on the surfaces of APCs to T cells. We first evaluated the cellular uptake of the Y-shape scaffold in DC2.4 cells. Upon treatment of cells for 3 h, confocal microscopy revealed the MEB fluorescence signal inside the cells, and most of the MEB fluorescence signal was colocalized with endolysosome (**Fig. 2A**). Note that free vaccines also showed high level of SIINFEKL antigen presentation, likely because in cultured cells, this minimal peptide antigen can directly bind with cell surface MHC molecules for antigen presentation without the requirement of intracellular delivery and processing. Next, we tested the ability of the above vaccines to elicit proinflammatory innate immune responses that drive T cell priming *in vitro*. We used a murine MHC-I-restricted model peptide antigen, SIINFEKL, which is derived from chicken ovalbumin (OVA). SIINFEKL was conjugated with DNA A3 as above (**Fig. S6**). We synthesized a Y-shaped DNA-scaffolded vaccine using CpG and SIINFEKL. Next, DC2.4 murine DC cells were treated with Y-shaped DNA-scaffolded vaccines for 24 h, with controls of PBS and admixed free CpG and free SIINFEKL, respectively. ELISA results showed that Y-shaped DNA-scaffolded vaccines elicited proinflammatory cytokine interleukin-6 (IL-6) (**Fig. 2B**). The proinflammatory immune responses are expected to promote the antigen presentation, potentiate the immunogenicity of the otherwise poorly immunogenic peptide antigens, and eventually elicit potent T cell responses. We further studied the presentation of SIINFEKL on DC2.4 cells treated as above by staining SIINFEKL/H-2K^b^ complexes on DC2.4 cell surfaces using an antibody. Flow cytometric analysis verified that the Y-shape DNA-scaffolded vaccines promoted the presentation of SIINFEKL on DCs (**Fig. 2C**). Consistently, treatment of DC2.4 cells with Y-shape DNA-scaffolded vaccines promoted these DCs to prime antigen-specific CD8^+^ T cells, as shown by using B3Z SIINFEKL-specific CD8^+^ T cell hybridoma (**Fig. 2D**).

### Delivery of AAco-AlbiVax to LNs and APCs

We then studied the delivery of these Y-shape DNA-scaffolded vaccines to draining LNs in mice. To do this, we conjugated Y-shape DNA-scaffolded vaccines with a near infrared dye IR800 which allows for the imaging of in cutaneous LNs in mice. Free IR800 and IR800 emulsified in a clinical benchmark IFA were used as controls. These formulations were subcutaneously (*s.c.*) administered at the tail base of BALB/c mice, which allow for lymphatic drainage to downstream inguinal LNs. By mouse whole-body IVIS imaging, we monitored the IR800 fluorescence intensities in inguinal LNs for up to 12 days post administration. We found that Y-shape DNA-scaffolded vaccines were accumulated in inguinal LNs significantly more than IFA-emulsified vaccines for as long as 7 days (**Fig. 2E, 2F**). The MEB-functionalized Y-shaped DNA scaffold showed the highest fluorescence intensity in draining LNs, indicating that the endogenous albumin hitchhiking by these scaffolds promoted LN homing and retention. The efficient delivery and durable retention of Y-shape DNA-scaffolded vaccines are expected to efficiently present antigens to APCs and elicit T cell responses in LNs.

### AAco-AlbiVax elicited robust T cell responses with memory

**Figure 3:**
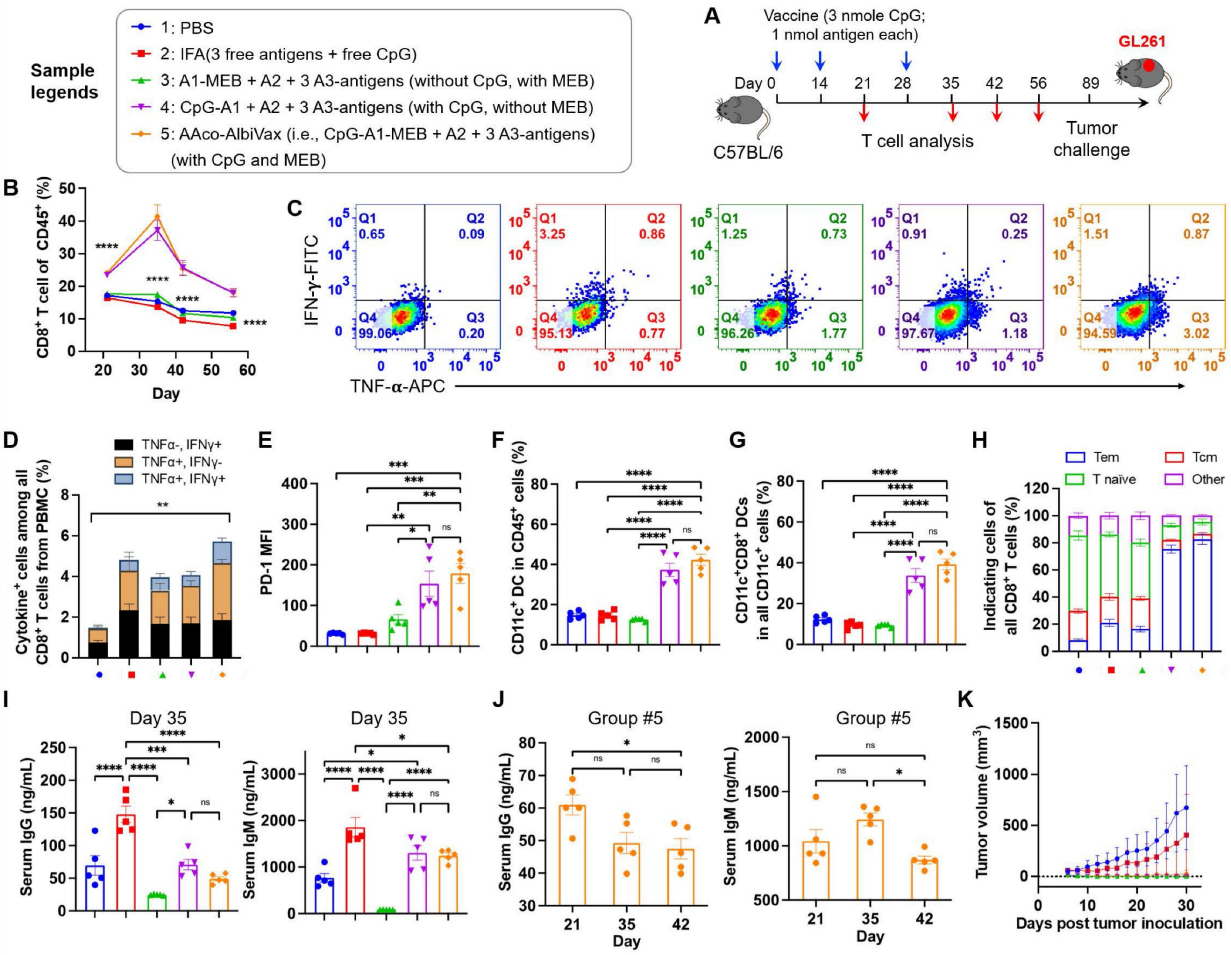
** GL261-specific neoantigen-based AAco-AlbiVax elicited T cell responses in mice.** (A) Study design of T cell response studies elicited by AAco-AlbiVax in C57BL/6 mice (*n* = 5). (B) Frequencies of CD8^+^ T cells in peripheral blood by flow cytometry over 56 days post priming. (C) Flow cytometry plots of cytokine^+^ in CD8^+^ T cells from peripheral blood, measured by intracellular staining of IFN-γ and TNF-α on day 35. (D) Intracellular cytokine staining results showing the percentages of cytokine^+^ CD8^+^ T cells on total PBMC CD8^+^ T cells on day 35. (E) MFI (Median Fluorescence Intensity) quantification of PD-1 levels on peripheral CD8^+^ T cells on day 35. (F, G) Representative flow cytometry quantification of CD45^+^CD11c^+^ DCs and CD8^+^ DCs (CD11c^+^CD8^+^B220^-^) in the above immunized mice on day 35. (H) Flow cytometry quantification of CD8^+^ memory T cells (central memory: CD62L^+^CD44^+^, effector memory: CD62L^-^CD44^+^ and naive T cells: CD62L^+^CD44^-^) in the above immunized mice on day 35. (I) ELISA results of serum anti-dsDNA IgG and IgM titers (day 35) in as-treated C57Bl/6 mice (n=5). AlbiCpG did not significantly induce anti-dsDNA IgG or IgM. (J) Serum anti-dsDNA IgG and IgM antibody titers for Group #5 over 42 days after priming. (K) Tumor growth curves in the above immunized mice challenged with GL261 on the right flank on day 89. Data: mean ± SEM; * *p* < 0.05; ** *p* < 0.01; *** *p* < 0.001 (one-way ANOVA).

Next, we studied the ability of Y-shape DNA-scaffolded GL261-specific neoantigen vaccines to elicit T cell responses in C57BL/6 mice. Specifically, mice were *s.c.* dosed with vaccines for three times with two-week intervals, followed by immune analysis such as T cell responses in peripheral blood at a series of days post immunization (**Fig. 3A**). As shown by flow cytometric analysis of PBMC, AAco-AlbiVax enhanced the frequencies of PBMC CD8^+^ T cells over at least 56 days post priming (**Fig. 3B, Fig. S7 B-D**). To study the antigen-specific T cell responses, we re-stimulated *ex vivo* PBMC CD8^+^ T cells with the corresponding three neoantigen peptides, followed by intracellular cytokine staining of IFN-γ and TNF-α. Flow cytometric analysis showed that AAco-AlbiVax enhanced the percentages of cytokine^+^ CD8^+^ T cells on CD8^+^ T cells (**Fig. 3C-D, Fig. S7**), indicating the ability of AAco-AlbiVax to elicit antigen-specific T cell responses in mice. We also observed an increased frequency of IFN-γ^+^TNF-α^+^ CD4^+^ T cells in the mice immunized with AAco-AlbiVax (**Fig. S7, 8A**). Moreover, the immunostimulation resulting from AAco-AlbiVax treatment enhanced the expression level of immune checkpoint PD-1 on peripheral CD8^+^ T cells (**Fig. 3E, Fig. S7**). This suggests the ability of AAco-AlbiVax to upregulate the immune checkpoint expression and thus sensitize these immune checkpoints for blockade, providing an opportunity for the rational combination of ICB with AAco-AlbiVax for the optimal tumor therapeutic efficacy. Further, AAco-AlbiVax enhanced the fraction of peripheral CD45^+^CD11c^+^ DCs and CD8^+^ DCs (CD11c^+^CD8^+^B220^-^) that are critical for the antigen cross presentation and the priming of antigen-specific CD8^+^ T cells (**Fig. 3F-G, Fig. S9**). On day 35, the frequency of CD45^+^CD11b^+^F4/80^+^ macrophages among PBMCs was not significantly changed by the above immunization (**Fig S8D and Fig. S9**). Antitumor immune memory is pivotal to prevent tumor recurrence for the long-term tumor therapeutic response. By flow cytometric analysis of PBMC CD8^+^ T cells stained for two immune memory phenotypic markers CD44 and CD62L, we showed that AAco-AlbiVax indeed enhanced the fractions of CD62L^+^CD44^+^ central memory and CD62L^-^CD44^+^ effector memory T cells (day 35) (**Fig. 3H, Fig, S8B**). To further verify the immune protection of immunization against the corresponding GL261 tumor cell growth, the above immunized mice were challenged with GL261 cells on the right flank on day 89. As a result, Y-shape DNA-scaffolded neoantigen vaccines completely prevented GL261 tumor growth, in contrast to moderate protection by IFA-emulsified peptide neoantigen vaccines (**Fig. 3K**). These results verified the potent GL261 neoantigen-specific T cell responses with long-lasting immune memory, which are critical for durable immunotherapy of established tumors with minimal tumor recurrence. Because extracellular DNA may elicit anti-DNA antibodies that cause autoimmune disorders, conjugating dsDNA scaffold with immunostimulatory adjuvant could induce vaccine-neutralizing anti-scaffold antibody response and adverse anti-DNA autoimmunity. As shown by ELISA results, even after two vaccine boosters, the scaffolded adjuvant elicited significantly less anti-dsDNA IgG and IgM, two primary anti-dsDNA antibody subtypes, than CpG emulsified in a clinical benchmark IFA (day 35) (**Fig. 3I, 3J**). Overall, these data support the promising safety of our vaccine delivery system.

### AAco-AlbiVax for GBM radioimmunotherapy

**Figure 4:**
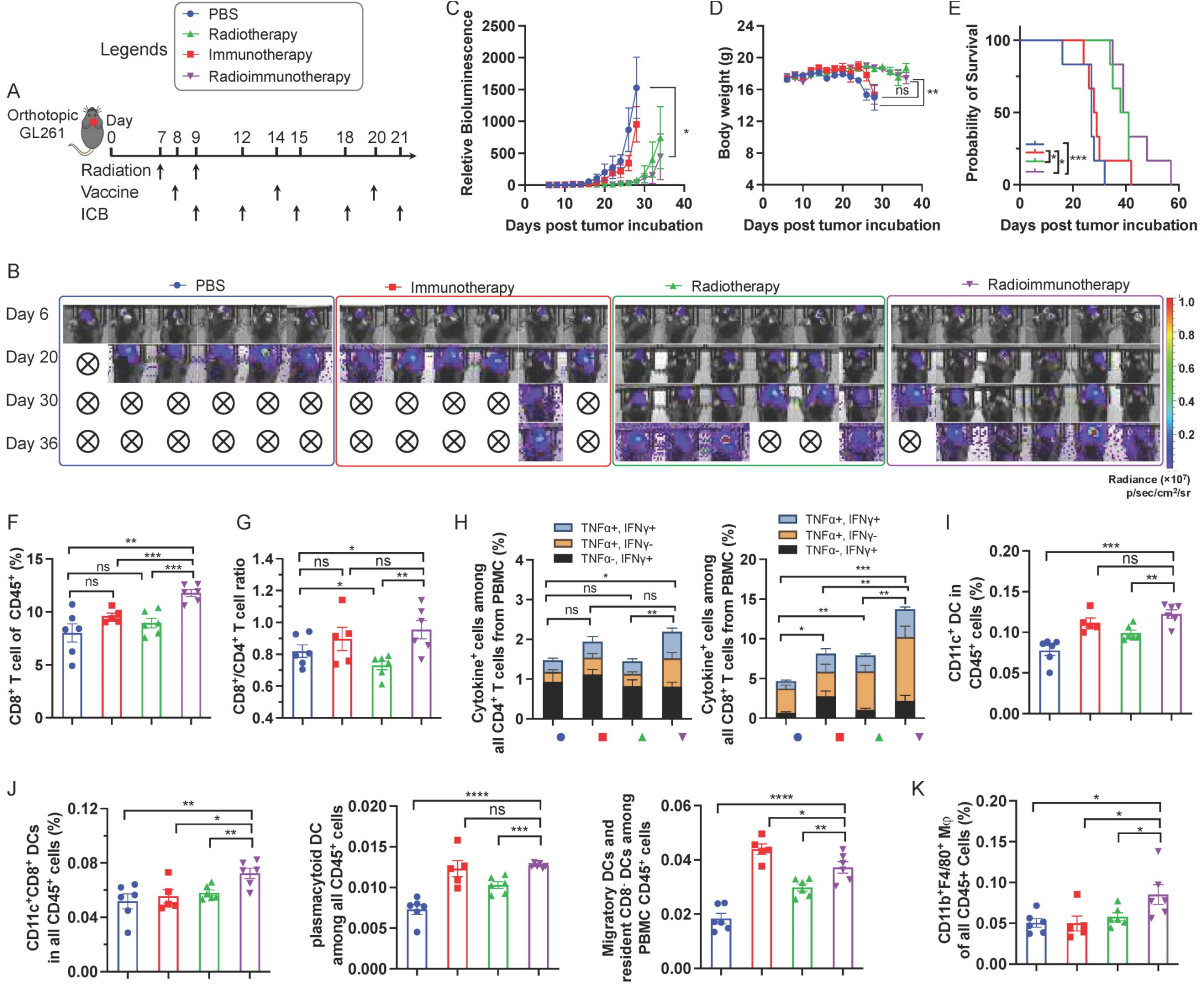
** Radioimmunotherapy of orthotopic GL261 GBM.** (A) Study design of radioimmunotherapy that combined AAco-AlbiVax, ICB, and radiotherapy for orthotopic GL261 in C57BL/6 mice (*n* = 6). αPD-1, αCTLA-4: 200 μg each, i.p. injection; vaccine: 3 nmole CpG, 1 nmol antigen each, s.c. injection at tail base. Radiation: 5 Gy. (B, C) Representative bioluminescence images and quantified tumor bioluminescence signal intensities in orthotopic GL261 GBM-bearing C57BL/6 mice. (D, E) and mouse body weight (D) and Kaplan-Meier overall survival curves (E) of the above treated mice. Asterisks in (E) indicates statistical analysis for radioimmunotherapy vs PBS, radioimmunotherapy vs immunotherapy, and radioimmunotherapy vs radiotherapy. (F, G) Frequencies of CD8^+^ T cells and CD8^+^/CD4^+^ T cell ratio in peripheral blood from the above treated mice by flow cytometry on day 27 after tumor inoculation. (H) Flow cytometry quantified percentages of cytokine^+^ CD4^+^ T cells and cytokine^+^ CD8^+^ T cells as measured by intracellular staining of IFN-γ and TNF-α on day 27. (I) Representative flow cytometry quantification of CD45^+^CD11c^+^ DCs among PBMCs of the above treated mice on day 27. (J) Representative flow cytometry quantification of major CD11c^+^ DC subsets of CD8^+^ DCs (CD11c^+^CD8^+^B220^-^), pDCs (CD11c^+^B220^+^) and migratory and residents CD8^-^ DCs (CD11c^+^CD8^-^B220^-^) among PBMCs of the above treated mice on day 27. (K) The percentage of CD45^+^CD11b^+^F4/80^+^ macrophages among PBMCs measured by flow cytometry on day 27. Data: mean ± SEM; * *p* < 0.05; ** *p* < 0.01; *** *p* < 0.001 (Student's t-test).

Encouraged by the ability of AAco-AlbiVax to elicit antigen-specific T cell responses, we then studied its therapeutic efficacy in orthotopic GL261 glioma-bearing C57BL/6 mice. For optimal therapeutic efficacy, we combined radiotherapy, with AAco-AlbiVax and dual ICB, the latter of which include antibodies against PD-1 and cytotoxic T-lymphocyte antigen 4 (CTLA-4). Radiotherapy is one of the current clinical standard-of-care treatment modalities for GBM, and is expected to reduce the tumor immunosuppression in addition to inducing tumor cell death. C57BL/6 mice (*n* = 6; 6-8 weeks) were stereotactically injected with luciferase/GFP-tagged GL261 cells (GL261-Luc) into the right striatum to establish orthotopic GL261 glioma. 7 days later, mice were randomly grouped to receive the following treatments, respectively: (1) PBS, (2) immunotherapy, in which mice received AAco-AlbiVax and ICB (αPD-1 + αCTLA-4), (3) radiotherapy (5Gy x 2, days 7 and 9), (4) radioimmunotherapy: mice received both radiotherapy and immunotherapy (AAco-AlbiVax + ICB). To initiate tumor cell killing and disrupt the immunosuppressive tumor microenvironment, radiotherapy was initiated on day 7 and repeated on day 9. Meanwhile, mice were subject to immunotherapy treatment by *s.c.* injection of AAco-AlbiVax at mouse tail base (days 8, 14, and 20) and intraperitoneal injection of ICB antibodies (days 9, 12, 15, 18, and 21) (**Fig. 4A**). Tumor burden was monitored by IVIS imaging of tumor bioluminescence signal. As shown in **Fig. 4B-C**, relative to PBS treatment, immunotherapy appears to slightly improve the tumor therapeutic efficacy; radiotherapy alone effectively controlled tumor growth at the early stage, and combination immunotherapy with radiotherapy slightly enhanced the tumor therapeutic efficacy relative to radiotherapy alone. This is consistent with the mouse body weight change, which is likely caused by mouse morbidity especially at the later stage of the study course (**Fig. 4D**), as well as mouse survival (**Fig. 4E**). Overall, immunotherapy showed moderate therapeutic efficacy, likely due to the very immunosuppressive tumor microenvironment of a relatively well-established glioma 8 days after tumor inoculation, which was supported by the combination of radiotherapy and immunotherapy significantly enhancing therapeutic efficacy by reducing the tumor immunosuppression. To investigate the underlying immunomodulation, we analyzed the peripheral immune profiles in the above mice on day 27. Relative to radiotherapy or immunotherapy alone, radioimmunotherapy significantly enhanced the CD8^+^ T cell frequencies among total PBMC CD8^+^ T cells (**Fig. 4F**). Radioimmunotherapy also enhanced the PBMC CD8^+^/CD4^+^ T cell ratio relative to radiotherapy, but not to immunotherapy alone (**Fig. 4G**). Intracellular staining of IFN-γ and TNF-α showed that radioimmunotherapy enhanced the percentages of not only cytokine-producing CD8^+^ T cells, but also cytokine-producing CD4^+^ T cells (**Fig. 4H**).

Moreover, immunotherapy and radioimmunotherapy enhanced the PBMC CD45^+^CD11c^+^ DCs, especially CD8^+^ DCs (CD11c^+^CD8^+^B220^-^), as well as pDCs (CD11c^+^B220^+^) and migratory and resident CD8^-^ DCs (CD11c^+^CD8^-^B220^-^) (**Fig. 4I, 4J**). Worth noting, relative to either immunotherapy or radiotherapy alone, radioimmunotherapy enhanced the frequencies of PBMC CD8^+^ DCs that are critical for antigen cross presentation. Finally, radioimmunotherapy enhanced the frequencies of PBMC CD45^+^CD11b^+^F4/80^+^ macrophages. Overall, these results suggest the ability of radioimmunotherapy to elicit multi-prone systemic antitumor immunity and show the potential for radioimmunotherapy to improve the tumor therapeutic efficacy relative to immunotherapy or radiotherapy alone.

## Conclusion

While current therapeutic modalities for GBM only have moderate therapeutic efficacy overall, radioimmunotherapy has the potential to synergistically combine the merits of radiotherapy and immunotherapy to improve GBM treatment outcome. Cancer therapeutic vaccines based on tumor neoantigen-specific personalized cancer vaccines hold great potential to elicit tumor-specific immunity. Here, we implemented AlbiVax platform and Y-shaped DNA scaffold for efficient codelivery of a TLR9 agonist with multivalent GBM GL261 neoantigen peptides. While many synthetic nanocarriers efficiently deliver vaccines,^54^ molecular vaccines are attractive to comply with current Good Manufacturing Practice (cGMP) in CMC for clinical application.^28^,^32^,^55^,^56^ Chemically-defined molecular AlbiVax^32^,^55^,^57^ bind to endogenous albumin to form nanocomplexes for efficient delivery, and thus leverage the merits of both molecular vaccines and nanovaccines while bypassing the complications associated with many synthetic nanocarriers. Albumin is stable (*t_1/2_*: ~20 days in humans) and abundant in the interstitium and lymphatics where vaccines are often administered.^58^ Albumin/AlbiVax nanocomplexes are efficiently taken up by APCs; in the acidic endosome, AlbiVax are released from albumin due to 20-fold weaker albumin-AlbiVax binding. Endosomal vaccine release allows 1) adjuvant CpG activation of TLR9 on endolysosome, and 2) MHC-I-restricted peptide neoantigen trafficking to the cytosol for antigen processing if needed, MHC complexation with antigenic epitopes, followed by antigen presentation on APC cell surfaces.^32^ Lastly, albumin receptor neonatal fragment crystallizable receptor (FcRn)^59^ is highly expressed in APCs^59^ and binds to albumin in acidic endosome to recycle albumin out of cells and prevent albumin degradation in lysosome.^60^,^61^ We further demonstrated the principle that AAco-AlbiVax potentiated the immunogenicity of these neoantigen peptides to elicit antitumor T cell responses while sensitizing immune checkpoint PD-1 for αPD-1 ICB therapy. Worth noting, despite the conjugation with an immunostimulant, the Y-shaped dsDNA did not elicit significant anti-dsDNA autoimmunity. Though AAco-AlbiVax only delivered three MHC-I-restricted antigens expected to elicit CD8^+^ T cells, the ability of radioimmunotherapy to elicit CD4^+^ T cell responses is likely due to the generation of endogenous MHC-II-restricted tumor antigens by radiation and the bystander effect of radioimmunotherapy. In an orthotopic GBM model, radioimmunotherapy significantly inhibited tumor progression, but the therapeutic efficacy did not significantly outperform that by radiotherapy alone; moreover, the therapeutic efficacy of immunotherapy, despite using a combination of trivalent AAco-AlbiVax and dual ICB agents (αPD-1 + αCTLA-4) did not significantly inhibited the growth of GL261 orthotopic glioma. This indicates that this tumor model is highly resistant to immunotherapy, and implies that to further enhance its immunotherapeutic efficacy, future studies need to further potentiate the antitumor immunity, broaden the antitumor T cell responses, reduce tumor immunosuppression, and test immunotherapy in alternative settings such as neoadjuvant therapy.

## Materials and methods

*Materials.* SMCC was purchased from TCI America. Mal-EB was synthesized as reported before.^32^ DNA CpG-A1-SS, A2-NH_2_ and A3-NH_2_ were purchased from Integrated DNA Technologies, Inc. The DNA sequences were shown in Table S1. Dithiothreitol (DTT) was purchased from Fisher Scientific, IFA from Sigma-Aldrich, and D-Luciferin sodium salt from Gold Bio.

*Synthesis of DNA oligonucleotide conjugates.* To get the CpG-A1-MEB, the CpG-A1-SS was reacted with DTT at 37 ºC for 4 hours in PBS. Then the mixture was purified with PD-10 column (GE Healthcare Life Sciences) following the instructions to remove extra DTT. Then Mal-EB was added and reacted at 37 ºC for 4 hours. The mixture was purified with PD-10 column to remove unbounded Mal-MEB and salt. The product of CpG-A1-MEB was lyophilized for storage. CpG-A1-MEB was characterized by HPLC using a gradient mobile phase of 0.1 M triethylammonium acetate (TEAA) in acetonitrile (buffer B) relative to 0.1 M TEAA in water (buffer A) from 15% to 60% buffer B over 15 min, then to 90% over 5 min. A3 modified with different peptide was obtained by first reacting A3-NH_2_ with SMCC. A3-NH_2_ was first reacted with SMCC by mixing with SMCC dissolved in DMF at 37 ºC for 4 hours and the solvent was evaporated by a rotatory evaporator to obtain the crude product. The crude product was dissolved in water and centrifuged at 12,000 rpm to remove excessive SMCC, then desalted using PD-10 columns to get the A3-SMCC. Then Ntrk1, Rtn2 and Imp3 was added to A3-SMCC solution respectively and reacted at room temperature overnight. The crude products were purified by HPLC using a gradient mobile phase of 0.1 M TEAA in acetonitrile (buffer B) relative to 0.1 M TEAA in water (buffer A) from 15% to 60% buffer B over 15 min, then to 90% over 5 min. The purified products were concentrated and desalted using PD-10 columns, then lyophilized for storage. A3-SIINFEKL was prepared as the other three peptides described. All the products were dissolved in nuclease-free water for storage in -80 ºC and the concentration were determined by Nanodrop (Thermo Scientific).

*Hybridization of the Y-shape scaffold.* The three Y-shape scaffolds were prepared by mixing same molar ratio of the CpG-A1-MEB, A2, and A3-Ntrk1 or A3-Rtn2 or A3-Imp3 in 1×TBE buffer with 12.5 mM MgCl_2_ respectively. The mixing solution were incubated at 95 °C for 10 min and slowly cooled to room temperature and keep it overnight for hybridization.

*Cells*. DC2.4 cells were cultured in RPMI 1640 medium. Luciferase/GFP-tagged GBM cell lines GL261 were cultured in DMEM medium. All medium was supplemented with 10% FBS and 0.1% penicillin and streptomycin. B3Z cells were cultured in complete RPMI 1640 medium with 2 mM L-glutamine, 1 mM sodium pyruvate, and 50 μM 2-mercaptoethanol. All cells were cultured in a humidified atmosphere (5% CO_2_, 37 °C) in a Biosafety Level II cabinet.

*In vitro* cell uptake. *In vitro* cell uptake was studied by confocal laser scanning microscopy (CLSM). DC2.4 cells were cultured on glass dishes and incubated with Y-shaped DNA scaffold modified with MEB (2.5 h). Then cells were stained with Hoechst 33342 and Lysotracker Green (Life Technologies) according to the manufacturers' instructions. 0.5 h later, cells were imaged on a Zeiss LSM 710 confocal microscope.

*Evaluation of APC activation and antigen presentation*. *In vitro* cytokine secretion and SIINFEKL presentation was evaluated in DC2.4 cells through ELISA, flow cytometry and B3Z assay. Specifically, cells were seeded in 96-well plates at a density of 3,000 cells per well. Then cells were treated with different formulations for 24 h. Then, supernatants were collected for cytokine detection by ELISA Kit (IL-6 from R&D Systems) following the manufacturer's instructions. To evaluate the cross-presentation of SIINFEKL by DC2.4 cells, flow cytometric analysis was performed for Ag presentation staining. DC2.4 cells were seeded on 24-well plates and treated with soluble CpG + SIINFEKL or the Y-shape vaccines. After incubation, DC2.4 cells were collected and stained with APC-labeled anti-SIINFEKL/H-2K^b^ antibody (BioLegend) for 0.5 hours on ice and then analyzed by flow cytometry (BD FACSCanto II). To evaluate Ag cross-priming from DC2.4 cells to T cells, a cell co-culture model comprised of DC2.4 cells and B3Z T cell hybridoma was performed. DC2.4 cells were seeded into 96-well plates and treated with different SIINFEKL formulations. After incubation, medium was aspirated, and DC2.4 cells were washed, then co-cultured with 10^4^ B3Z cells for another 24 h. Then cells were lysed for 4 h at 37 °C with lysis buffer (PBS with 100 mM 2-mercaptoethanol, 9 mM MgCl_2_, 0.2% Triton X-100 and 0.15 mM CPRG). The reaction was stopped by 1 M sodium carbonate. The magnitude of Ag priming was evaluated through absorbance measurements (λ = 570 nm).

*Animals.* All animal work was conducted following NIH guidelines and in accordance with an approved protocol by the Virginia Commonwealth University Animal Care and Use Committee (IACUC). Female C57BL/6 mice (6-8 weeks) were purchased from Charles Rivers Laboratories.

*In vivo NV delivery to draining LNs.* To evaluate LN delivery, infrared dye (IR800) labeled CpG-A1were used for hybridization. PBS, IFA loaded IR800-CpG-A1 were used as control. Different formulation solution was *s.c.* injected at the tail base. Whole-body image was measured after 6 h, and once a day until day 12 by IVIS imaging (Caliper Life Sciences).

*In vivo immunization and Ag-specific T cell response.* The three antigens formulated Y-shape scaffold were used to study *in vivo* T cell responses. C57BL/6 mice were immunized via *s.c.* injection at the base of the tail with different formulations of vaccines on days 0 and 14: (1) PBS, (2) IFA-emulsified CpG and three antigens, (3) hybridization of A1-MEB, A2 with A3-Ntrk1, A3-Rtn2, a3-Imp3 respectively, (4) hybridization of CpG-A1-MEB, A2 with A3-Ntrk1, A3-Rtn2, a3-Imp3 respectively, (5) hybridization of CpG-A1, A2 with A3-Ntrk1, A3-Rtn2, a3-Imp3 respectively, (6) hybridization of A1-MEB, A2, with A3-Ntrk1, A3-Rtn2, and A3-Imp3 respectively. (7) hybridization of CpG-A1-MEB, A2, with A3-Ntrk1, A3-Rtn2, and A3-Imp3 respectively. On day21, peripheral blood cells were collected, and red blood cells were lysed using ACK lysis buffer (BioLegend) for 5 min and removed by centrifugation. Cells were washed twice in PBS and stained using Zombie Aqua (BioLegend). Then cells were suspended in cold PBS supplemented with 0.1% FBS and stained with a dye-labeled staining cocktail including CD8α-APC/Cy7, CD44-Alexa Fluor 647, CD62L-FITC, and PD-1-Brilliant Violet 421 (BioLegend). Then cells were washed, and resuspended in Cytofix (BioLegend) for 20 min at 4°C. For intracellular staining of INFg-FITC and TNFa-APC, cells were permeabilized and washed with Perm/Wash buffer twice under instructions (BioLegend, 426803) after fixing. Then cells were stained with intercellular antibodies and then were washed twice, resuspended for flow cytometry using a BD LSRFortessa-X20.For antigen present cells staining, after using Zombie Aqua (Biolegend) for live/dead staining, we used the cocktail of CD45-Brilliant Violet 421, CD11c-Alexa Fluor 594, CD11b-PE/Cy5, CD8α-PE, F4/80- APC/Cy7 and B220-FITC. On day 28, mice were vaccinated with different formulations again. On day 34, tumor challenge was conducted by *s.c.* inoculation with GL-261 tumor cells (2 × 10^6^) on the right shoulder. Tumor sizes and body weight were monitored every 3 days. Mice were euthanized if the total tumor volume exceeded 2,000 mm^3^. Tumor volume was calculated as Volume = (length × width × width)/2.

*Combination immunotherapy of orthotopic GBM.* Female C57BL/6 mice (6-8 weeks) were stereotactically injected with 25,000 Luciferase/GFP-tagged GBM cells (GL261-Luc) into the right striatum using a 22-gauge Hamilton syringe with the following coordinates: +1.00 mm anterior, 2.5 mm lateral, and 3.00 mm deep to establish brain tumors. On day7, mice were randomly divided into four groups (n = 6): (1) PBS, (2) immunotherapy: mice received vaccine + ICB (αPD-1 + αCTLA-4), (3) radiotherapy: mice received radiotherapy only, (4) radioimmunotherapy: mice received radiotherapy + vaccine + ICB. Mice received radiotherapy on day 7 and day 9. Vaccine treatment were start on days 8, 14 and 20. ICB were administered intraperitoneally on days 9, 12, 15, 18, and 21. The bioluminescence signal was observed by an IVIS 10 min after the injection of 100 μL luciferin (30 mg/mL) per mouse.

*Peripheral immune analysis.* Mouse peripheral T cell and APC responses were stained for analysis. Peripheral blood was collected from the above treated mice on day 27. Red blood cells were lysed using ACK lysis buffer (BioLegend) for 5 min and removed by centrifugation. Cells were washed twice in PBS and stained using Zombie Aqua (BioLegend). Then cells were suspended in cold PBS supplemented with 0.1% FBS and stained with a dye-labeled staining cocktail including CD8α-APC/Cy7, CD44-Alexa Fluor 647, CD62L-FITC, and PD-1-Brilliant Violet 421 (BioLegend). Then cells were washed, and resuspended in Cytofix (BioLegend) for 20 min at 4°C. For intracellular staining of INF-g-FITC and TNF-a-APC, cells were permeabilized and washed with Perm/Wash buffer twice under instructions (BioLegend, 426803) after fixing. Then cells were stained with intercellular antibodies and then were washed twice, resuspended for flow cytometry using a BD LSRFortessa-X20. For APC staining, after using Zombie Aqua (Biolegend) for live/dead staining, we used the cocktail of CD45-Brilliant Violet 421, CD11c-Alexa Fluor 594, CD11b-PE/Cy5, CD8α-PE, F4/80- APC/Cy7 and B220-FITC for staining.

## Supplementary Material

Supplementary figures and table.
