# Supplementary material for: Lymph node-targeting adjuvant/neoantigen-codelivering vaccines for combination glioblastoma radioimmunotherapy
Source: Theranostics. 2023 Aug 6;13(13):4304–15. doi: 10.7150/thno.84443 (PMC10465217; doi:10.7150/thno.84443)
Supplement: Supplementary file 1 — Supplementary figures and table. [file thnov13p4304s1.pdf]

Supporting Information

Lymph node-targeting adjuvant/neoantigen-codelivery vaccines for combination glioblastoma radioimmunotherapy

Ting Su[1], Shurong Zhou[1,6], Suling Yang [1,6], Nicholas Humble[2], Fuwu Zhang[3], Guocan Yu[4], Paula D. Bos[5], Furong Cheng[1,*], Kristoffer Valerie[2], Guizhi Zhu[1,6*]

[1] Department of Pharmaceutics and Center for Pharmaceutical Engineering and Sciences; The Developmental Therapeutics Program, Massey Cancer Center; Virginia Commonwealth University, Richmond, VA 23298, USA

[2] Department of Radiation Oncology, School of Medicine; The Developmental Therapeutics Program Program, Massey Cancer Center; Virginia Commonwealth University, Richmond, VA 23298, USA

[3] Department of Chemistry, University of Miami, Coral Gables, FL 33146, USA

[4] Key Laboratory of Bioorganic Phosphorus Chemistry & Chemical Biology, Department of Chemistry, Tsinghua University, Beijing, 100084, China

[5] Department of Pathology, School of Medicine; Cancer Biology Program, Massey Cancer Center; Virginia Commonwealth University, Richmond, VA 23298, USA.

[6] Department of Pharmaceutical Sciences, College of Pharmacy; Biointerfaces Institute. University of Michigan, Ann Arbor, MI 48109, USA

* Corresponding author: F.C. (chengf2@vcu.edu), G.Z. (email: guizhiz@umich.edu)

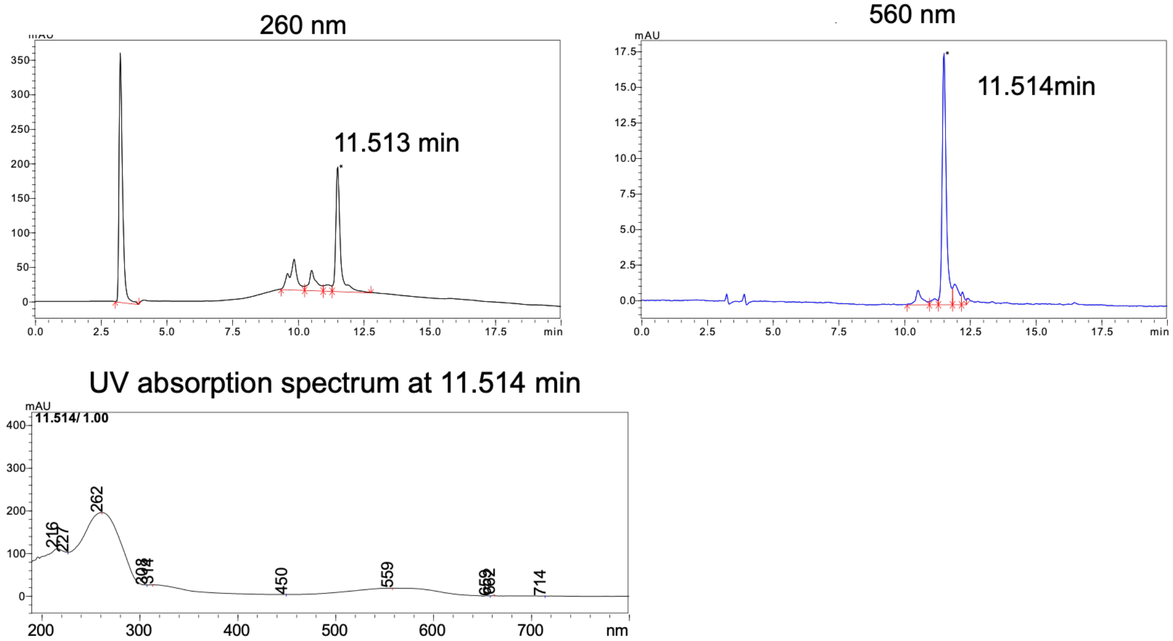

Figure S1: HPLC chromatograms and UV absorption of CpG-A1-MEB. CpG-A1-MEB showed both the characteristic peaks for DNA and MEB at 11.51 min of retention time, indicating successful MEB conjugation with CpG-A1.

A

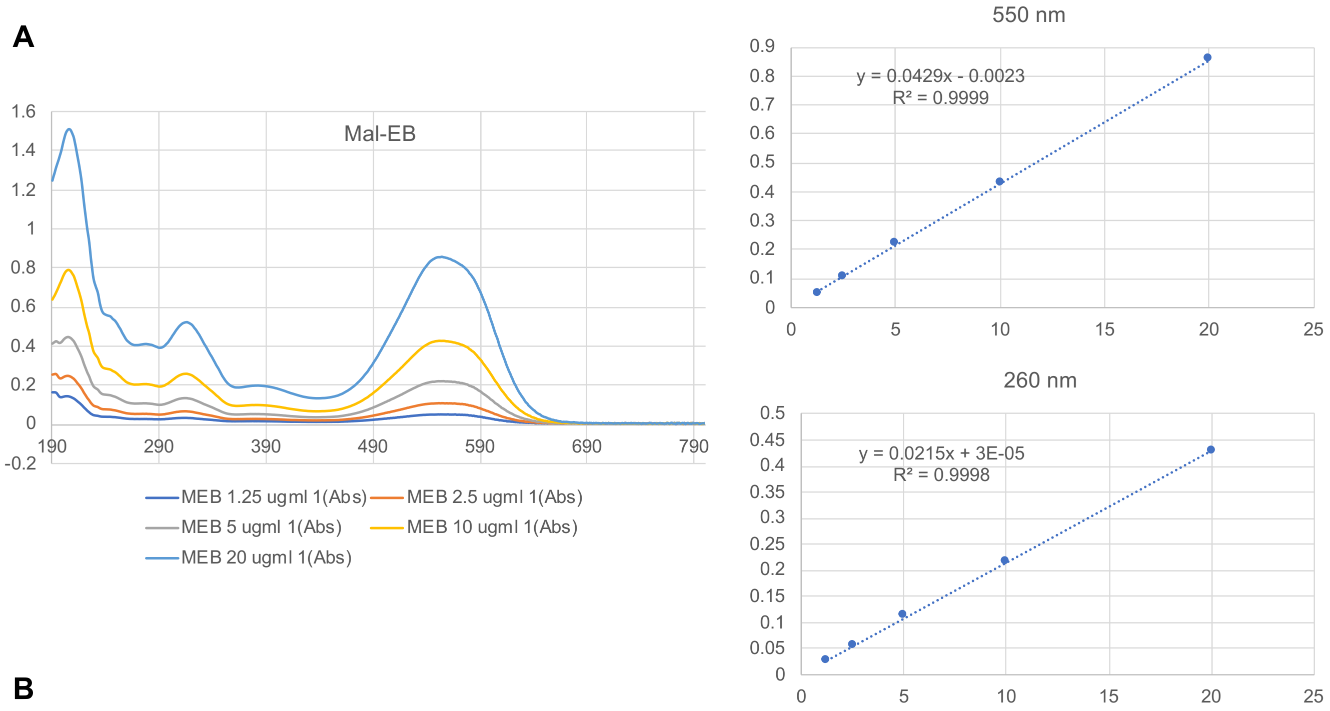

B

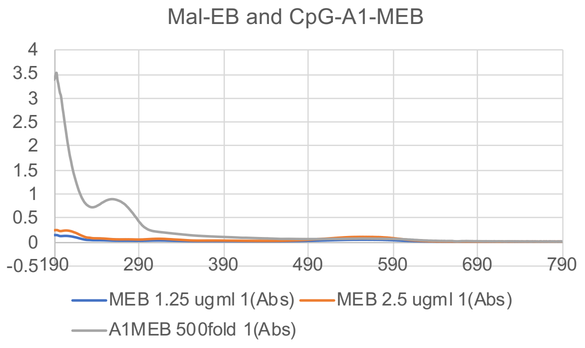

	A260	A550
CpG-A1-MEB (dilute 500x)	0.89888	0.08278
MEB (1.9832 ug/ml)	0.04267	0.08278

Figure S2: (A) The UV–Vis absorption spectrum of MEB at different concentration and the standard curve from 550 nm to 260 nm. (B) Absorption of free MEB (1.25 or 2.5 mg/mL) and CpG-A1-MEB (after 500x dilution of its stock stolution) (For CpG-A1-MEB, A260 = 0.8988 and A550 = 0.08278). Using standard curve of MEB absorption at 550 nm vs concentration, the MEB concentration in the CpG-A1-MEB was calculated to be 1.9832 mg/mL. At this concentration, the absorption of free MEB at A260 is 0.04267. By comparing the UV absorption of CpG-A1-MEB with that of free MEB at 1.25 mg/mL or 2.5 mg/mL, the influence of MEB absorption at 260 nm on CpG-A1-MEB absorption at 260 nm is very small.

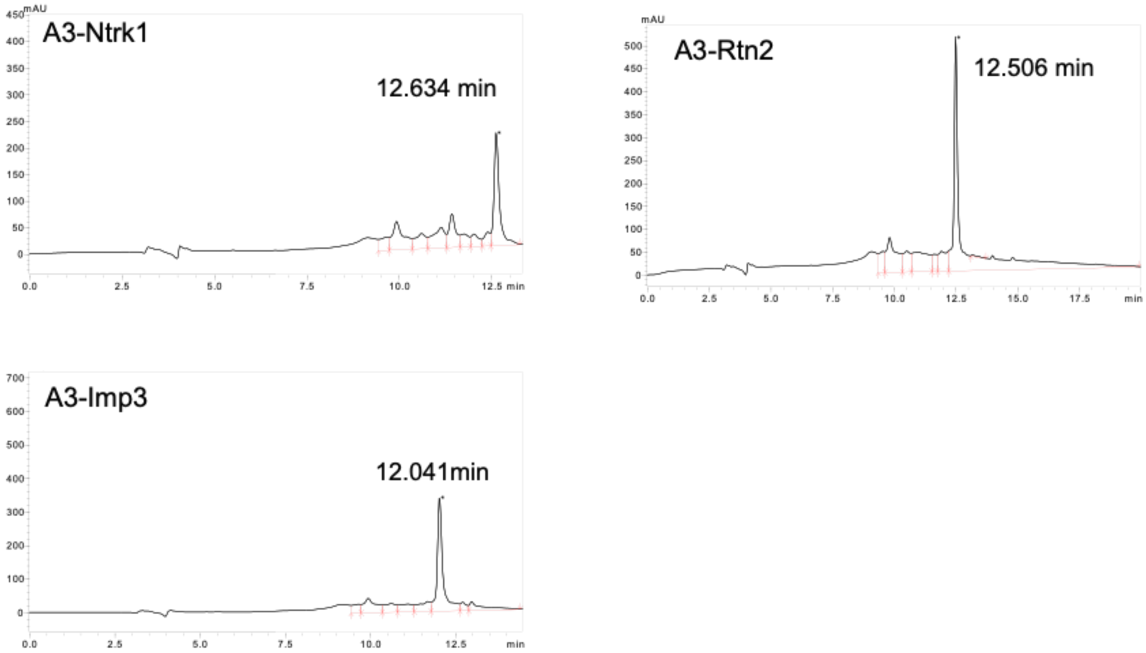

Figure S3: HPLC chromatograms of A3-Ntrk1, A3-Rtn2 and A3-Imp3.

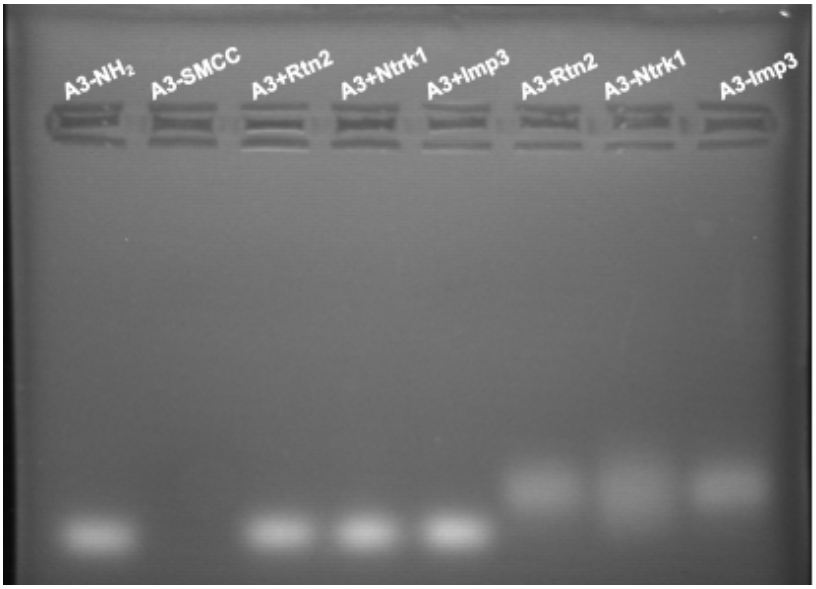

Figure S4: Image of agarose gel electrophoresis showing the products of A3-Rtn2, A3-Ntrk1 and A3-Imp3, free mixing of A3 with peptide Rtn2, Ntrk1 and Imp3 are used as control (A3+Rtn2, A3-Ntrk1 and A3-Imp3).

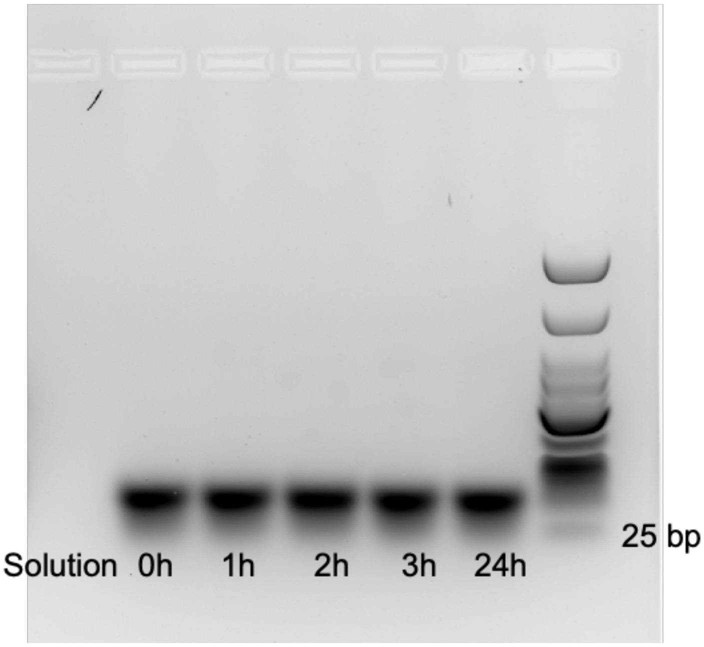

Figure S5. Stability of Y-shaped DNA in PBS containing 10% FBS (Solution) at 37 °C for 24 hours by gel electrophoresis.

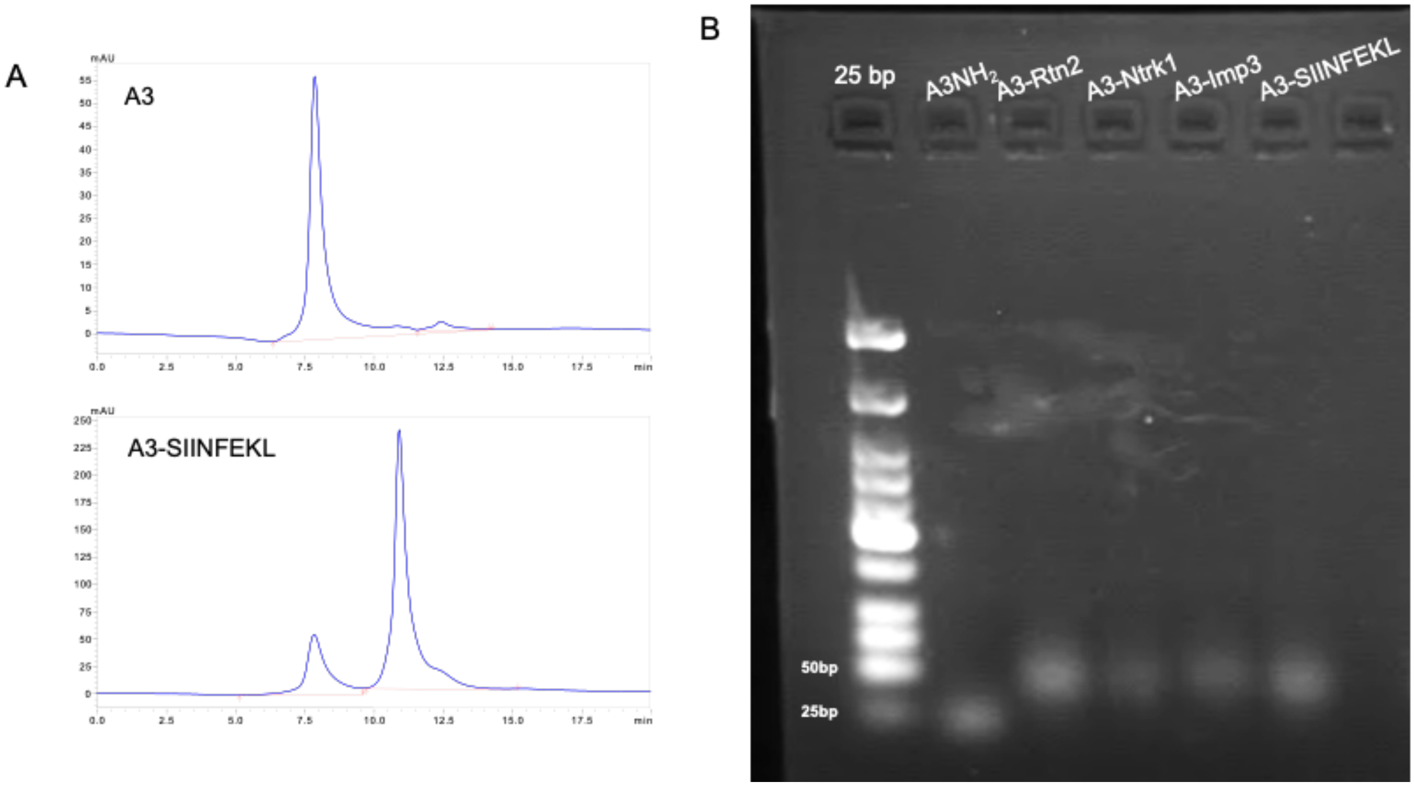

Figure S6: Synthesis of A3-SIINFEKL conjugate. (A) Representative HPLC chromatograms of A3 and A3-SIINFEKL. (B) Image of agarose gel electrophoresis showing the products of A3-Rtn2, A3-Ntrk1, A3-Imp3 and A3-SIINFEKL.

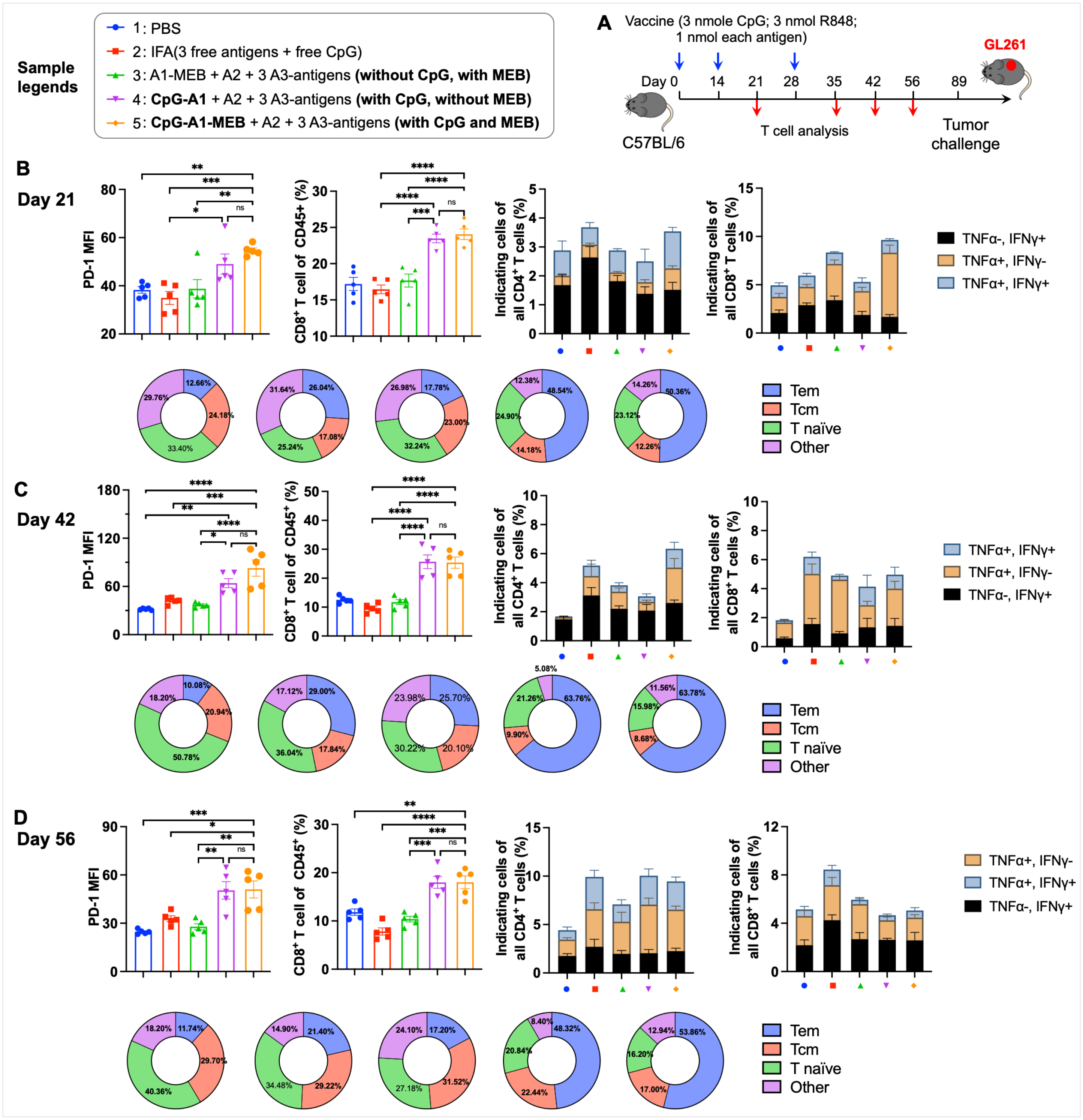

Figure S7. Analysis of T cell responses elicited by AAco-AlbiVax in mice. (A) Study design of AAco-AlbiVax T cell responses in C57BL/6 mice (*n* = 5), the same study shown in Figure 3. (B-D) Flow cytometry data showing

the peripheral T cell responses elicited by AAco-AlbiVax as above on Days 21, 42 and 56, including MFI of PD-1 expression on peripheral CD8^{+} T cells, CD8^{+} T cell percentages, percentages of cytokine^{+} T cells among CD8^{+} T cells and CD4^{+} T cells, and quantification of CD8^{+} memory T cells (Central memory: CD62L^{+}CD44^{+}, effector memory: CD62L^{-}CD44^{+} and naive T cells: CD62L^{+}CD44^{-}). Data: mean ± SEM; * $p < 0.05$; ** $p < 0.01$; *** $p < 0.001$ (Student's t-test).

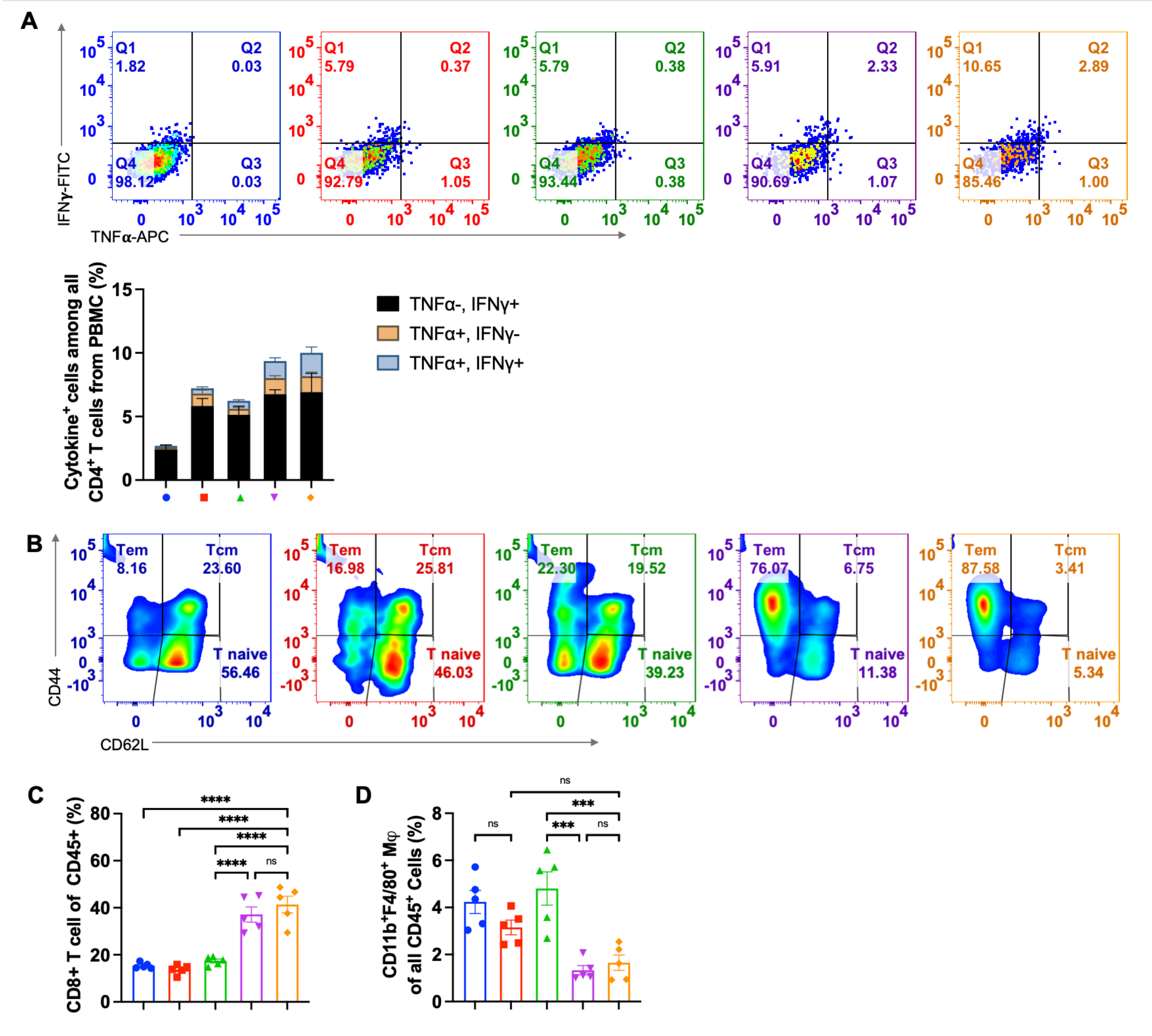

Figure S8. Flow cytometric analysis of peripheral blood from the above immunized mice (Figure S6, Figure 3) on day 35. (A) Intracellular cytokine staining results showing the percentages of cytokine^{+} CD4^{+} T cells on total PBMC CD4^{+} T cells. (B) Flow cytometry plots of CD8^{+} memory T cells (central memory: CD62L^{+}CD44^{+}, effector memory: CD62L^{-}CD44^{+} and naive T cells: CD62L^{+}CD44^{-}). The quantified data are shown in Figure 3H. (C) Frequencies of CD8^{+} T cells in peripheral blood by flow cytometry at day 35. (D) Quantification of CD45^{+}CD11b^{+}F4/80^{+} macrophages in peripheral blood by flow cytometry at day 35.

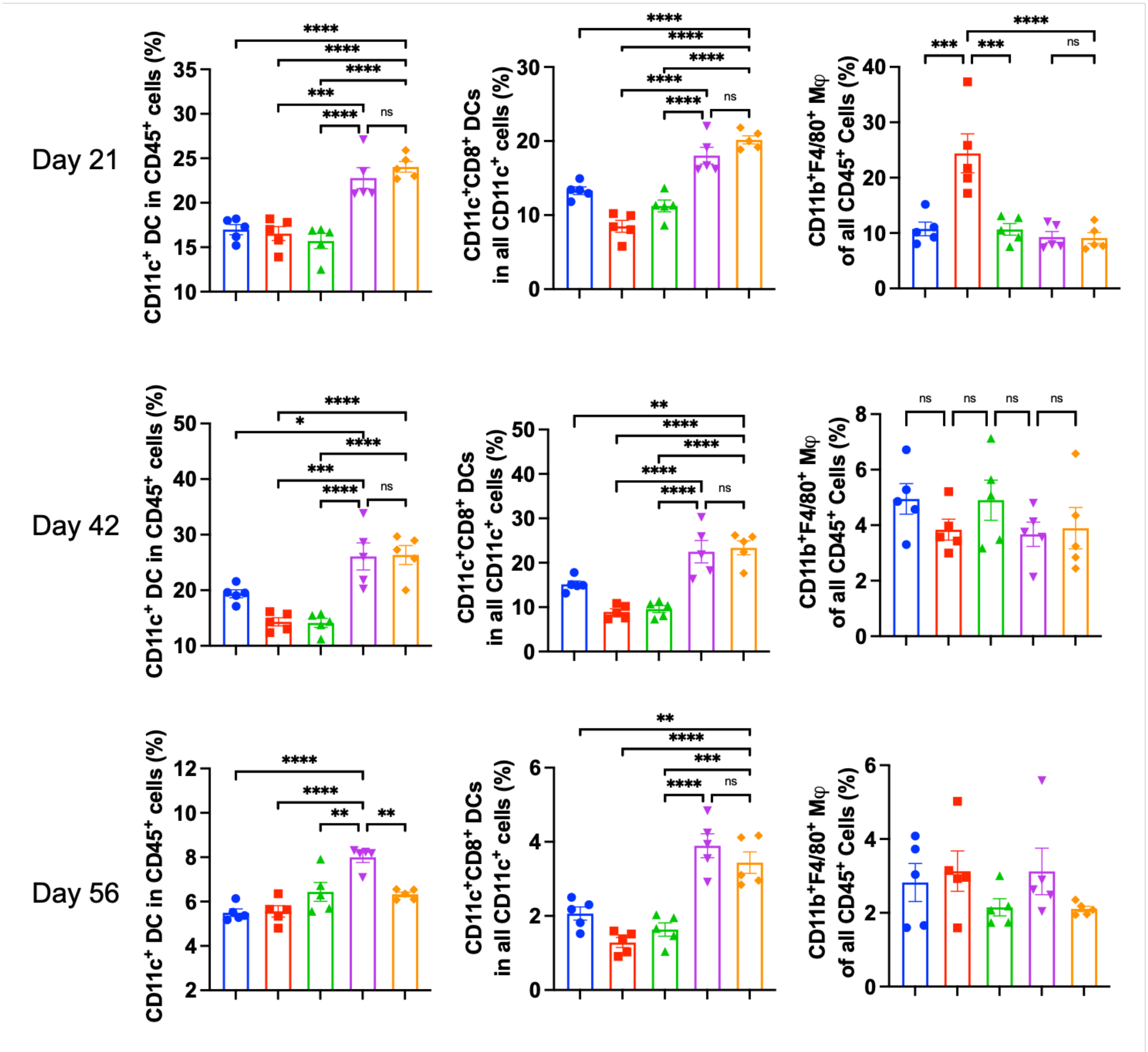

Figure S9. Quantification of $CD45^{+}CD11c^{+}$ DCs, $CD8^{+}$ DCs ($CD11c^{+}CD8^{+}B220^{-}$) and $CD45^{+}CD11b^{+}F4/80^{+}$ macrophages among PBMCs from the immunized mice as shown in Figure S6 or Figure 3 (Day 35). Data: mean ± SEM; * $p < 0.05$; ** $p < 0.01$; *** $p < 0.001$ (Student's t-test).

Table S1. DNA sequences.

	Sequences (5' - 3')
CpG	TCCATGACGTTCCTGACGTT
A1	TTTTT GAGTCAGTCACTGTGTCGTG
CpG-A1	TCCATGACGTTCCTGACGTT TTTTT GAGTCAGTCACTGTGTCGTG - SH
A2	TTTTT CGAGAACCTATGACTGACTC
A3	NH_2 - TTTTT CACGACACAGTAGGTTCTCG
